# Activation of HIV-1 from Latent Infection via Synergy of RUNX1 Inhibitor Ro5-3335 and SAHA

**DOI:** 10.1371/journal.ppat.1003997

**Published:** 2014-03-20

**Authors:** Zachary Klase, Venkat S. R. K. Yedavalli, Laurent Houzet, Molly Perkins, Frank Maldarelli, Jason Brenchley, Klaus Strebel, Paul Liu, Kuan-Teh Jeang

**Affiliations:** 1 Laboratory of Molecular Microbiology, National Institute of Allergy and Infectious Diseases, National Institutes of Health, Bethesda, Maryland, United States of America; 2 Host Virus Interaction Branch, National Cancer Institute, National Institutes of Health, Bethesda, Maryland, United States of America; 3 Genetics and Molecular Biology Branch, National Human Genome Research Institute, National Institutes of Health, Bethesda, Maryland, United States of America; University of Massachusetts Medical School, United States of America

## Abstract

A major barrier to the elimination of HIV-1 infection is the presence of a pool of long-lived, latently infected CD4+ memory T-cells. The search for treatments to re-activate latent HIV to aid in clearance is hindered by the incomplete understanding of the mechanisms that lead to transcriptional silencing of viral gene expression in host cells. Here we identify a previously unknown role for RUNX1 in HIV-1 transcriptional latency. The RUNX proteins, in combination with the co-factor CBF-β, are critical transcriptional regulators in T-cells. RUNX1 strongly modulates CD4 expression and contributes to CD4+ T-cell function. We show that RUNX1 can bind DNA sequences within the HIV-1 LTR and that this binding represses transcription. Using patient samples we show a negative correlation between RUNX1 expression and viral load. Furthermore, we find that pharmacologic inhibition of RUNX1 by a small molecule inhibitor, Ro5-3335, synergizes with the histone deacetylase (HDAC) inhibitor SAHA (Vorinostat) to enhance the activation of latent HIV-1 in both cell lines and PBMCs from patients. Our findings indicate that RUNX1 and CBF-β cooperate in cells to modulate HIV-1 replication, identifying for the first time RUNX1 as a cellular factor involved in HIV-1 latency. This work highlights the therapeutic potential of inhibitors of RUNX1 to re-activate virus and aid in clearance of HIV-1.

## Author Summary

Since it was first discovered in the early 1980s, Human Immunodeficiency Virus 1 (HIV-1), the causative agent of Acquired Immunodeficiency Syndrome (AIDS), has been the focus of intense research. In untreated individuals, the number of CD4+ T-cells in the blood slowly drops over time and the patient eventually succumbs to an opportunistic infection. Although current therapies are capable of managing the virus; they do not represent a true cure. As a retrovirus, HIV-1 incorporates itself into the host genome and survives in the long-lived population of memory T-cells found in the human host. In this study, we examine the roll of a T-cell specific transcription factor (RUNX1) in the control of HIV-1 replication. Through various molecular studies, we show that RUNX1 represses HIV-1 replication in T-cells. By examining samples from patients with HIV-1, we are able to show a negative correlation between viral replication and RUNX1 expression. Finally, we show that an inhibitor of RUNX1 synergizes with Vorinostat, a current lead compound in the quest to re-active HIV-1 and purge the latent pool.

## Introduction

Human Immunodeficiency Virus type I (HIV-1) is the etiologic agent of Acquired Immunodeficiency Syndrome (AIDS). HIV-1 has a complex life cycle that in part involves a unique transcriptional interaction between the viral Tat protein and its target RNA element (TAR) found in the R sequence of the LTR [1]–[3]. In the absence of treatment, most HIV-1 infected individuals will experience a steady decline in the number of CD4+ T-cells, progress to AIDS and eventually die as the result of acquiring opportunistic infections.

Transcriptional control of HIV-1 occurs in two phases. Basal transcription of the integrated provirus first occurs at a low level in a Tat independent manner [4]. Once the Tat protein is synthesized, viral transcription transits to a Tat-dependent route. Tat binds TAR RNA and recruits a complex of cyclin T1 and CDK9 to the start site of transcription [1] leading to the phosphorylation of the c-terminal domain (CTD) of the RNA Pol II to induce more processive transcription. Tat has also been shown to help initiate transcription through interaction with the TATA Binding Protein as well as various histone modifying enzymes such as CBP/p300 and the PBAF complex [5].

The HIV-1 LTR contains a myriad of transcription factor-binding sites, such as those for SP1 and NF-κB. It is believed that interactions of cellular factors with the HIV-1 LTR determine active transcription versus the establishment of transcriptional latency. HIV-1 latency, a state in which the infected cell produces little to no viral RNA, represents a major barrier to viral eradication in an infected individual [6]–[8].

In mammals, there are three RUNX proteins that can interact with a cofactor, core-binding factor β (CBF-β), to form an active transcription factor complex [9], [10]. RUNX protein binding to CBF-β allows transport of the complex into the nucleus via a localization signal in the RUNX protein [11]. In turn, CBF-β increases the affinity of RUNX proteins for DNA. This complex is essential for proper differentiation of cells of the hematopoietic lineage. Of particular interest is the involvement of RUNX1 in the differentiation and fate selection of CD4+ T-cells [12]–[15]. Specifically, RUNX1 is drastically down regulated when thymocytes progress from double-negative to double-positive during development, and it is also down-regulated when naïve CD4+ T-cells are stimulated through the T-cell receptor (TCR) to become effector cells. In the latter case, RUNX1 down-regulation is associated with increased IL-2 production which is likely critical for CD4+ T-cell function [14].

Mechanistically, RUNX serves a role in the initiation of transcription that is likely achieved through p300 histone acetyltransferase recruitment. Intriguingly, RUNX family members may also recruit repressive factors such as mSin3A, Suv39H1 and histone deacetlyases and serve transcriptional repressor functions. Indeed, RUNX1 is important in repressing CD4 expression in double-negative thymocytes and mature CD8+ T-cells. The choice to act as an activator or repressor is influenced by post-translational modifications of the RUNX protein in the core binding factor [16], [17] and through the recruitment of selective factors to a specific promoter [18], [19].

Three recent publications have highlighted a role for CBF-β in Vif function [20]–[22]. Specifically, CBF-β is capable of binding to Vif and this interaction increases Vif mediated APOBEC3G degradation. A recent study has presented evidence that CBF-β binding by Vif influences RUNX responsive genes [23]. These studies suggest that the Vif protein has evolved to bind CBF-β. The presumption made in the first two studies is that this mechanism is specific to protection of the virus from APOBEC3G. However, the follow-up study suggests the intriguing possibility that Vif may be binding to CBF-β in order to influence RUNX mediated gene expression.

Our current work suggests that RUNX1 may be an important HIV-1 LTR-binding factor that serves a role in latency. RUNX1, as a T-cell specific transcription factor capable of suppression or activation of target promoters, may alter the transcription of the viral LTR during cellular infection. We find that inhibition of RUNX1 by a small molecule inhibitor synergizes with the HDAC inhibitor SAHA to activate HIV-1 latency. Our findings indicate that RUNX1 with its binding partner CBF-β may act to repress LTR transcription and show that this repression might be countered by the viral Vif protein.

## Results

### RUNX1 and CBF-β over-expression reduce expression of viral proteins and viral replication

**Figure 1:**
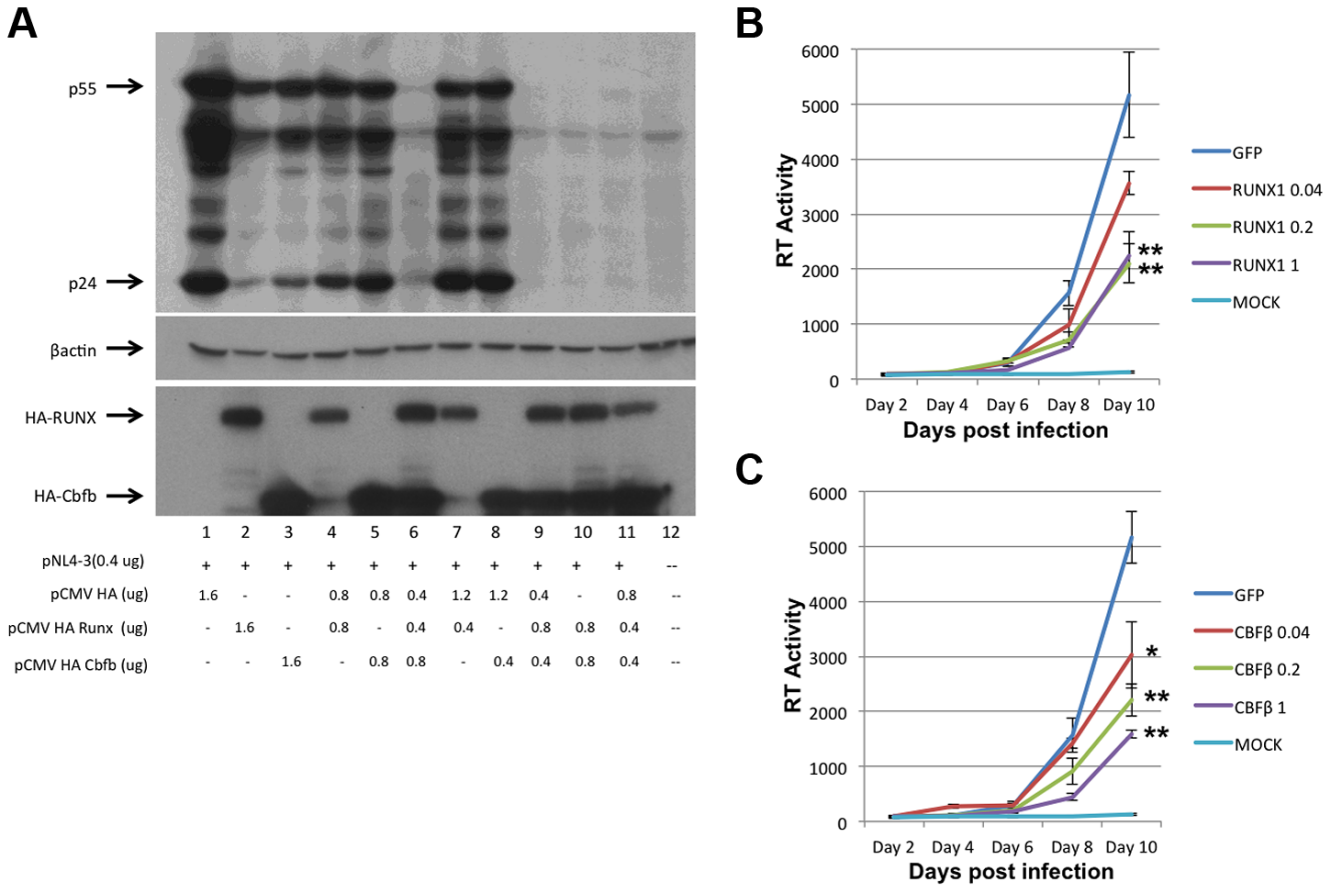
RUNX1 and CBF-β are capable of repressing HIV-1 replication. **A**) HeLa cells were transfected with the pNL4-3 proviral plasmid and the indicated amounts of HA-tagged RUNX1 or CBF-β expression vectors. Forty-eight hours post transfection cells were harvested, protein extracts were prepared and extracts were Western blotted for p24 and p55 Gag using human immune serum (upper panel), β-actin (middle panel) or HA (lower panel). Jurkat T-cells were transfected with1 ug of pMAXGFP or 0.04, 0.2 or 1 ug of **B**) pRUNX1 or **C**) pCBF-β and infected with NL4-3 twenty-four hours post transfection. Viral replication was followed by RT at days 2, 4, 6, 8 and 10 post infection.

To assess the involvement of RUNX1 in HIV-1 transcription, RUNX1 and CBF-β expression plasmids were co-transfected with an HIV-1 molecular clone (pNL4-3) into HeLa cells (Fig 1A). Twenty-four hours post transfection, cells were harvested and protein extracts were prepared and subjected to Western blot analysis of HIV-1 protein expression. Interestingly, the transfection of either RUNX1 or CBF-β alone reduced HIV Gag expression in a dose dependent manner (Fig. 1A, compare lanes 2+3, 4+5 and 7+8 to lane 1). Transfection of RUNX1 and CBF-β together reduced Gag expression to nearly undetectable levels (Fig. 1A, lanes 6, 9, 10 and 11).

We next tested the effect of RUNX1 and CBF-β on spreading HIV-1 infection of Jurkat T-cells. In brief, Jurkat cells were transfected with 0.04, 0.2 or 1 ug of pMaxGFP control, pRUNX1 or pCBF-β expression vector using Amaxa nucleofection. Observation of GFP expression in transfected control cells showed that the transfection efficiency was >75%. Western blot analysis was performed to confirm over-expression of CBF-β and RUNX1 (Supplementary Figure S1). Twenty-four hours after transfection, cells were infected with the NL4-3 virus and cell culture supernatants were sampled at 2, 4, 6, 8 and 10 days post infection. Quantification of reverse transcriptase activity (RT) in the culture supernatants showed typical HIV-1 spreading infection in the cells transfected with the control vector (Figure 1B, C). However, increasing the transfected amounts of either RUNX1 (Fig. 1B) or CBF-β (Fig. 1C) produced reduced HIV-1 replication; with RT levels repressed to 43% and 30% of the control on day 10. Conversely, knock down of RUNX1 or CBF-β expression by ∼60% in a cell line model of latency induced a two-fold increase in RT production (Supplementary Figure S2). Taken together, these data are consistent with an important role served by RUNX1 and CBF- β in HIV-1 replication.

### RUNX1 and CBF-β repress HIV-1 transcription

**Figure 2:**
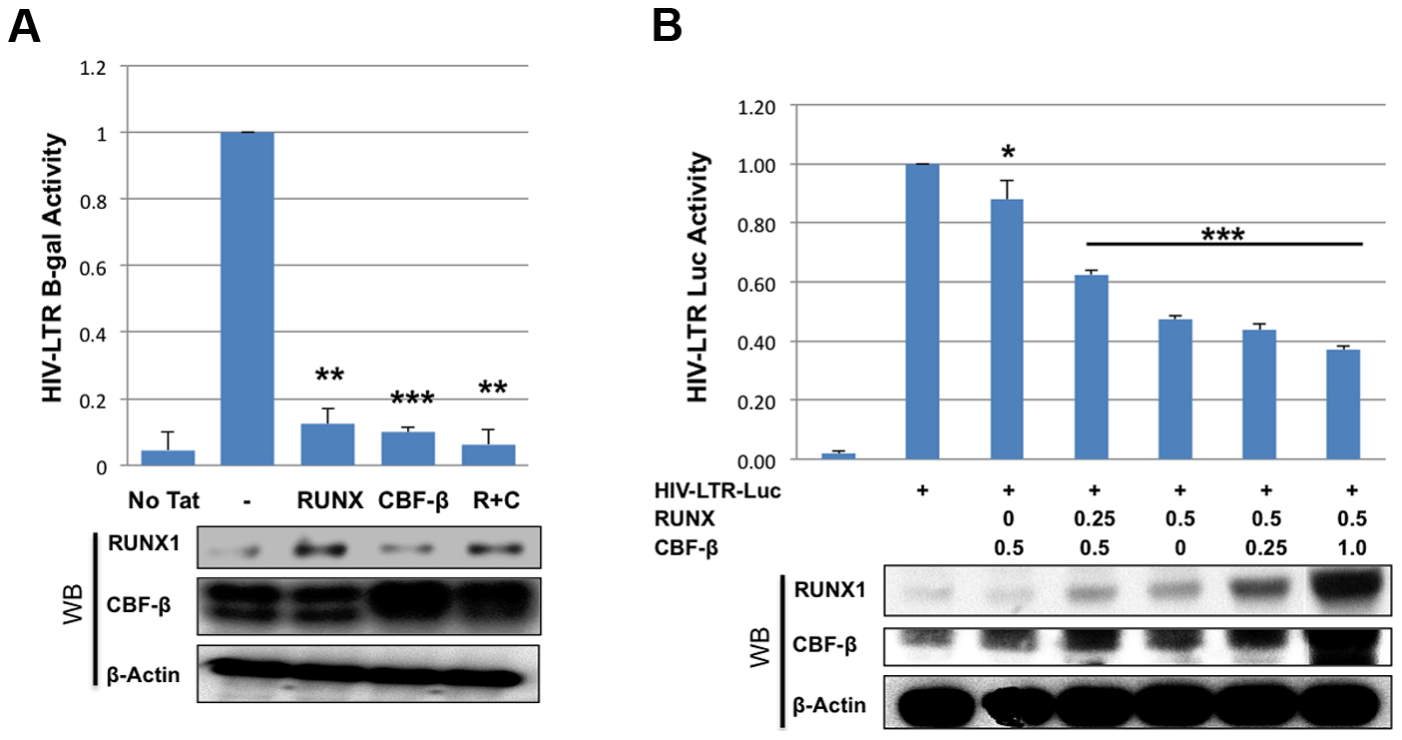
RUNX1 and CBF-β suppress LTR-driven promoter expression of the integrated and unintegrated LTR. **A**) TZMbl indicator cells were seeded in a six well plate and transfected with 0.01 μg Tat expression vector and 1 μg of RUNX1 or CBF-β expression vectors or a combination of the two. Forty-eight hours after transfection cells were lysed and β-galactosidase activity was measured by a luminescence assay. β-galactosidase is represented as relative to transfection of Tat alone. **B**) 293T cells were seeded in a 6-well plate and transfected with pLTR-GL3 and RUNX1 or CBF-β vectors as indicated. Forty-eight hours after transfection the cells were harvested and cell lysates prepared. Cell extracts were then used to determine the production of luciferase. Results are displayed as luciferase signal relative to transfection of reporter construct alone. Western blotting was performed on transfected cells to assess the levels of RUNX1 and CBF-β expression. * p≤0.05, ** p≤0.01 and *** p≤0.001

To further characterize the effects of RUNX1 and CBF-β on the transcription of an integrated HIV-1 LTR, we used the TZMbl reporter cell line. TZMbl cells are derived from HeLa cells to express CD4 and HIV-1 entry co-receptors, CXCR4 and CCR5; they contain an integrated HIV-1 LTR driving expression of firefly luciferase and a second integrated HIV-1 LTR driving the beta-galactosidase reporter gene. TZMbl were transfected with a Tat expression vector, together with RUNX1 or CBF-β expression vectors or a combination of the two. Forty-eight hours after transfection, the cells were harvested and equal protein amounts were used to determine β-galactosidase activity (Fig. 2A). We observed that the transfection of one microgram of either RUNX1 or CBF-β expression vector suppressed LTR-driven β-galactosidase expression by 88% and 90% respectively. Transfecting both vectors together repressed LTR-driven β-galactosidase expression by 94%.

We also tested the effect of RUNX1 and CBF-β on the basal activity of the HIV-LTR in 293T cells. 293T cells were transfected with 1 ug pLTR-GL3 (an HIV LTR driven luciferase reporter), 0.5 ug of RUNX1 or CBF-β expressing plasmid and increasing amounts of CBFβ or RUNX1 respectively (Fig. 2B). Transfection of CBF-β alone only modestly repressed LTR-driven transcription (Fig. 2B, 88% of control, 3^rd^ column). Cotransfecting 0.25 ug of RUNX1 plasmid further repressed reporter expression to 62% of baseline (Fig. 2B, 4^th^ column). Transfection with RUNX1 alone reduced reporter activity to 47% of control (Fig. 2B, 5^th^ column). Increasing the levels of transfected CBF-β further reduced activity to a modest degree (Fig. 2B, compare columns 5 to 6 and 7). RUNX1 and CBF-β over-expression had no effect on either CMV or EF1 control promoter constructs and were capable of activating a murine leukemia virus promoter known to be responsive to RUNX1 (BXH2 LTR) (Supplementary Figure S3). Taken together these data demonstrate that RUNX1 and CBF-β are capable of suppressing the promoter activity of integrated or un-integrated HIV-1 LTRs, which is consistent with our earlier findings [24].

### The HIV-1 LTR contains potential RUNX binding sites

**Figure 3:**
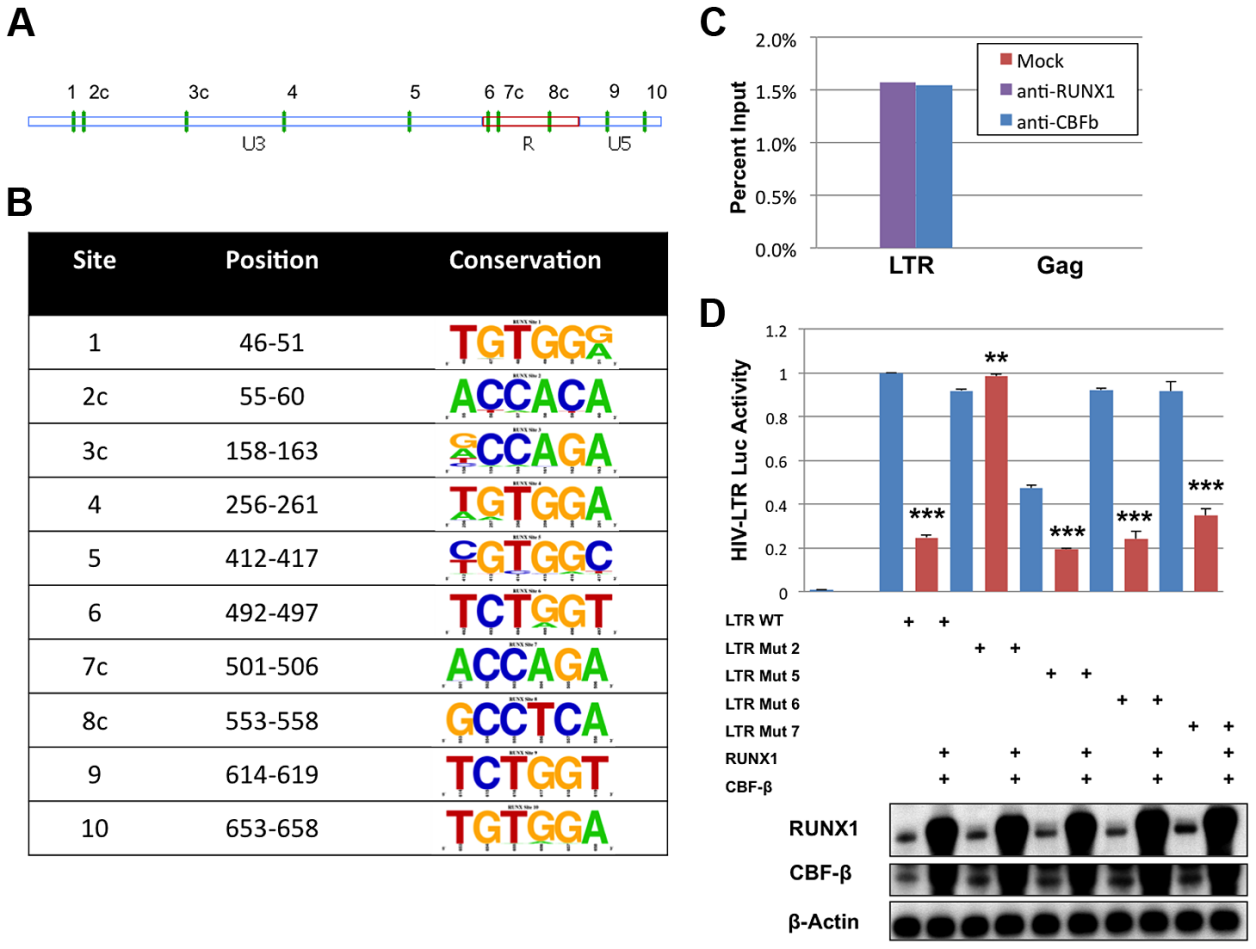
RUNX1 binds to the HIV-1 LTR. **A**) The position of the ten predicted binding sites (green vertical dashes) represented graphically on the LTR. The letter ‘c’ following the number indicates orientation on the reverse strand. **B**) The sequences corresponding to the potential binding sites were imported into UC-Berkley's WebIcon program and used to generate graphical representations of conservation. The height of each letter in the vertical column represents the percentage of sequences that contain that base at that position. Positions where only one letter is visible represent 100% conservation within the 58 sequences examined. **C**) HIV-1 infected ACH2 T-cell line was treated with 1% formaldehyde, lysed and then sonicated to shear chromatin to ∼1000 bp. Sheared chromatin was incubated with antibodies against RUNX1, CBF-β or no antibody control. Antibody bound chromatin complexes were immuno-precipitated with protein A/G agarose beads and DNA was detected by qPCR against the LTR or Gag sequence. **D**) 293T cells were seeded in a 6-well plate and transfected with pLTR-GL3 WT, mutant 2, mutant 5, mutant 6 or mutant 7 and RUNX1 or CBF-β vectors as indicated. Forty-eight hours after transfection the cells were harvested and cell lysates prepared. Cell extract were then used to determine the production of luciferase. Results are displayed as luciferase signal relative to transfection of the wild-type promoter construct. Western blotting was performed on transfected cells to assess the levels of RUNX1 and CBF-β expression. ** p≤0.01 and *** p≤0.001

The DNA binding site, consensus sequence TGYGGT [25], for the RUNX proteins has been described for several promoters including *MHC I, KIR*, the BXH2 LTR and *RUNX1* itself [25]–[28]. In order to identify potential binding sites in the HIV-1 LTR the TransFac matrix of RUNX1 binding sites was used to search the DNA sequence of the NL4-3 provirus from U3 through Gag using a search algorithm available from the University of Pennsylvania [29]. This analysis revealed 10 potential binding sites in the LTR and none in the 5′ portion of the Gag coding region. Of these 10 sites, six exist in the positive orientation, while four reside on the complementary strand (Fig. 3A and 3B). The conservation of these potential sites was analyzed using fifty-eight clade B sequences that contain a complete 5′LTR from the Los Alamos HIV Sequence Database (Fig. 3B) [30]. This analysis revealed that sites 3, 4 and 5 are not well conserved amongst clade B viruses. However, the remaining seven sites are well conserved. Indeed, these sequences appear to be better conserved than other regions of the LTR, suggesting that they may be selected for function.

Chromatin immuno-precipitations (ChIP) were performed to evaluate binding of RUNX1 to the potential LTR sites. It is known that CBF-β binds to RUNX1 and increases its affinity for DNA; thus, our ChIP analysis was performed in the context of both proteins. Chromatin was isolated from the HIV-1 latently infected ACH2 T-cell line, sheared by sonication, and immunoprecipitated with antibodies for RUNX1, CBF-β or no antibody control. The association of RUNX1 or CBF-β with HIV-1 LTR DNA or Gag DNA as a negative control was measured by qPCR (Fig 3C). ChIP analysis revealed the binding of RUNX1 and CBF-β to the LTR but not Gag DNA.

To specifically identify RUNX1 binding sites within the LTR, mutant reporter constructs were generated. Site-directed mutagenesis was used to change the final two nucleotides (consensus GT will be changed to TG) in potential RUNX binding sites 2, 5, 6 and 7 (Fig. 3A, B). This alters the important fifth residue in the binding site that has previously been shown to abrogate RUNX binding [31]. The four mutant reporter constructs were then used to evaluate RUNX1 responsiveness in 293T cells, which were transfected with 1 μg reporter (pGL3-LTR WT, pGL3-LTR mut2, pGL3-LTR mut5, pGL3-LTR mut6 and pGL3 mut7), 0.5 μg of CBF-β expression plasmid and 0.5 μg of RUNX1 expression vector. Forty-eight hours after transfection, the cells were harvested, lysed and 20 μg of protein extract used for luciferase assay (Figure 3D). RUNX1 and CBF-β expression repressed WT LTR to 25% of control (compare columns 1 and 2). Mutants 5, 6 and 7 were also repressed by RUNX1 and CBF-β. However, mutant 2 was not repressed by RUNX1 and CBF-β expression (compare columns 3 and 4). This suggests that the predicted binding site 2 is a physiological RUNX1 binding site in the LTR.

### Vif expression relieves RUNX1 and CBF-β repression of HIV-1 transcription

**Figure 4:**
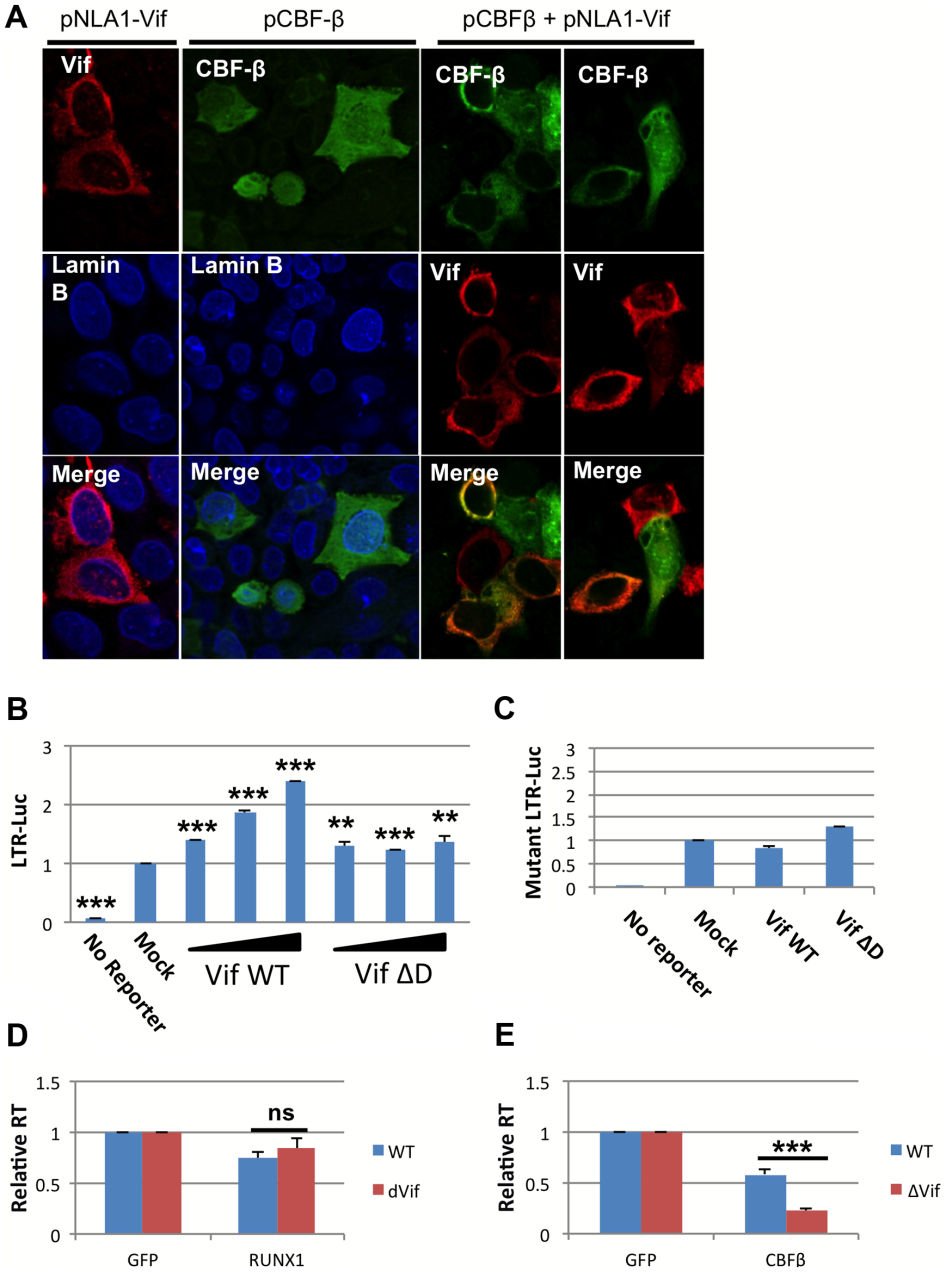
HIV-1 Vif sequesters CBF-β in the cytoplasm. **A**) HeLa CBF-β knockdown (KD) cells (5×10^6^) were cotransfected with the Vif expression vector pNL-A1 (2.5 μg) and/or the CBF-β vector pCBF-β (1 μg). Total amounts of transfected DNA were adjusted to 5 μg using empty vector DNA as appropriate. Three hours after transfection, cells were trypsinized and seeded onto cover slips. Cells were fixed 24 hr later in methanol (10 min, -20°C) and then stained with rabbit antibodies to Vif (Vif93; 1:100) or CBF-β (Thermo Fisher; 1:100). Bound antibodies were visualized by Texas-Red or Cy2-conjugated secondary antibodies (Jackson Labs; 1:100). Images were collected on a Zeiss LSM410 confocal microscope using a Plan-Apochromat 63x/1.4 oil immersion objective (Zeiss). **B**) 293T cells were seeded in a 6-well plate and transfected with 0.1 ug pLTR-GL3 and 0.04, 0.2 and 1 ug pVif or pVif ΔD. Forty eight hours post transfection cells were collected and used to prepare protein extracts. Cell extracts were then used to determine the production of luciferase. Results are displayed as luciferase signal relative to transfection of reporter construct alone. **C**) 293T cells were seeded in a 6-well plate and transfected with 0.1 ug pLTR-GL3 mutant 2 and 1 ug of pVif or pVif ΔD. Luciferase activity was determined as above. Jurkat cells were transfected with GFP plasmid and **D**) RUNX1 or **E**) CBF-β plasmid and infected twenty-four post transfection with equivalent doses of NL4-3 WT or NL4-3 ΔVif. RT values were determined at days 2, 4, 6, 8 and 10 post infection. Data is presented as RT activity relative to control (GFP transfected) cells. ** p≤0.01 and *** p≤0.001

Recently it was shown that HIV-1 Vif can bind to CBF-β and that this interaction increases APOBEC3G degradation [20]–[22]. The ability of Vif, a cytoplasmic protein, to bind CBF-β may alter the ability of CBF-β to function as a cofactor for the RUNX proteins. CBF-β itself has no nuclear localization signal. Instead, it relies on binding to a RUNX protein partner to be ferried into the nucleus [11]. We wondered if binding of CBF-β by Vif may sequester CBF-β in the cytoplasm. To test this hypothesis, Vif and CBF-β expression vectors were transfected alone or in combination into HeLa cells (Fig 4A). 24 hours after transfection, the cells were stained for Vif, CBF-β and the nuclear marker Lamin B. Vif alone localized to the cytoplasm; and CBF-β alone was seen throughout the cell (Panel A, 2^nd^ column), which is consistent with prior studies [11]. However, in cells that over expressed both Vif and CBF-β (Panel A, 3^rd^ and 4^th^ column), CBF-β was localized entirely in the cytoplasm consistent with the notion that Vif sequesters CBF-β in the cytoplasm.

Based on the published association of Vif with CBF-β and the above data, we explored Vif expression as a model for CBF-β depletion. 293T cells were transfected with 0.1 μg LTR-luciferase reporter and Vif expression vector (Fig 4B). Luciferase activity at forty-eight hours post-transfection was determined. Transfection of increasing amounts of Vif led to increased luciferase expression. Vif binds CBF-β through its n-terminal region, and mutation of residues 21 and 38 in Vif disrupts this binding [20], [22]. To test that Vif binding to CBF-β is required to mediate the observed increase in LTR expression, we utilized a vector that expresses a Vif protein lacking residues 23–43 (Vif ΔD). Transfection with Vif ΔD showed a small, but not dose responsive, increase in LTR driven luciferase expression.

To further confirm the involvement of Vif in rescuing RUNX1 mediated repression of the LTR, we employed the LTR mutant 2 reporter construct that was demonstrated above (Fig 3D) to be un-responsive to RUNX1 or CBF-β overexpression (Fig 4C). Transfection of neither Vif WT nor Vif ΔD was able to substantially increase Luciferase expression from this vector, supporting that the Vif effect on the LTR involves RUNX1 and CBF-β.

The ability of Vif to counteract repression mediated by RUNX1 and CBF-β in a spreading infection was monitored using a ΔVif virus. Jurkat cells were again used as they have been classified as ‘permissive’ due to their lack of APOBEC3G expression and ability to support the replication of viruses lacking Vif [32], [33]. Jurkat cells were transfected with 0.04 μg pRUNX1 or pCBF-β (Fig 4D and E). Twenty-four hours post-infection cells were infected with NL4-3 or NL4-3 ΔVif. Reverse transcriptase activity at day 10 demonstrated no difference in the susceptibility of the viruses to RUNX1 overexpression (Panel D). However, NL4-3 ΔVif was significantly more sensitive to CBF-β overexpression (Panel E). CBF-β repressed WT virus to 58% of the control, while NL4-3 ΔVif was repressed to 23% of the control. This data confirms that Vif is able to counteract CBF-β repression of HIV infection.

### RUNX1 expression in CD4+ memory T-cells of viremic HIV-1 patients correlates negatively with viral load and positively with CD4+ T-cell count

In order to determine the relevance of RUNX1 expression in HIV-1 infection in human patients we examined the expression of RUNX1, CBF-β and RUNX3 in primary T-cells. Analysis of CD4+ T-cell populations (Supplementary Figure S4) shows that upon activation of naïve CD4+ T-cells (a population relatively refractory to infection) the expression levels of RUNX1, RUNX3 and CBF-β drop (correlating with greater susceptibility to infection). Interestingly, the expression of these proteins in naïve cells is fairly uniform, but expression in the memory pool (the primary target cells during infection) is much more variable. This person-to-person variability leaves open the possibility that RUNX1 expression levels may correlate with clinical outcomes.

**Figure 5:**
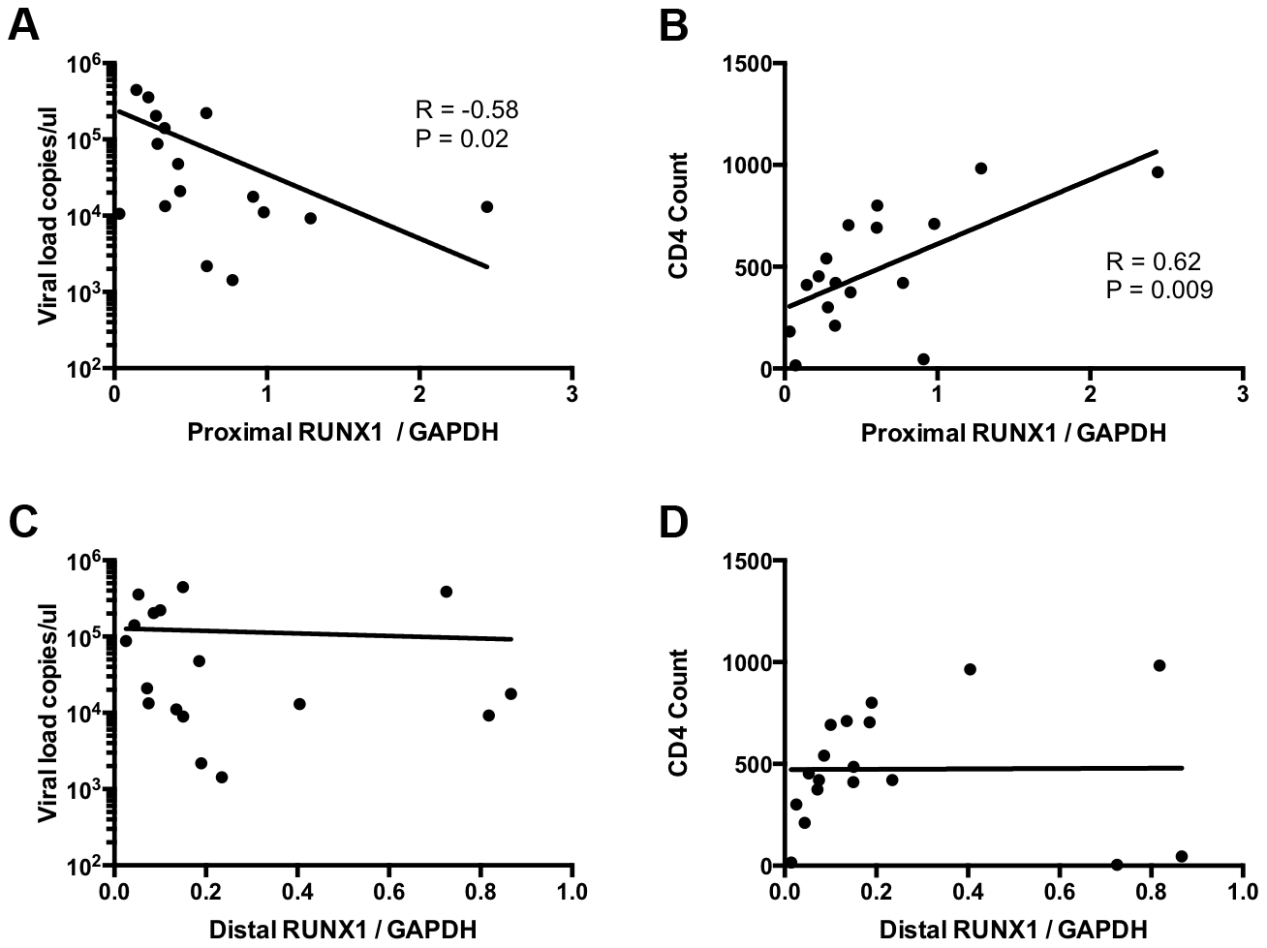
RUNX1 expression in memory CD4+ T-cells correlates with clinical metrics in patients. CD4+ memory T-cells were sorted from total PBMC of viremic HIV-1 patients in the absence of therapy. RNA extract from these cells was used to measure the expression of promoter proximal and distal RUNX1 as normalized to GAPDH. Expression level were then graphed as follows: **A**) promoter proximal RUNX1 vs viral load, **B**) promoter proximal RUNX1 versus CD4+ T-cell count, **C**) promoter distal RUNX1 versus viral load and **D**) promoter distal RUNX1 versus CD4+ T-cell count. R and P values were computed using Spearman test.

To determine the relevance of RUNX1 expression in HIV-1 infection in human hosts we compared RUNX1 expression to viral load and CD4+ T-cell counts in viremic HIV-1 patients who were not on therapy. Previous studies have shown that two promoters drive RUNX1 expression: a proximal and distal promoter. Each promoter codes a transcript that varies in the 5′ coding region and studies suggest that one isoform may be more important than the other in terms of function [34], [35]. In keeping with this, no significant correlation was seen between total RUNX1 levels and either viral load or CD4+ T-cell counts (Data not shown). However, when examining the specific levels of the promoter proximal RUNX1 transcript in memory CD4+ T-cells we noted a significant negative correlation with viral load and a significant positive correlation with CD4+ T-cell count (Fig 5A and 5B). This was in contrast to promoter distal RUNX1 transcripts that showed no correlation with viral load or CD4+ T-cell count (Fig 5C and 5D). Memory CD4+ T-cells are the primary target of infection and account for the majority of virus seen in the blood of patients. Therefore, these findings suggest the intriguing possibility that RUNX1 expression may have a role to play in the clinical progression of HIV-1 in patients.

### An inhibitor of RUNX1:CBF-β interaction synergizes with SAHA to reactivate latent HIV

**Figure 6:**
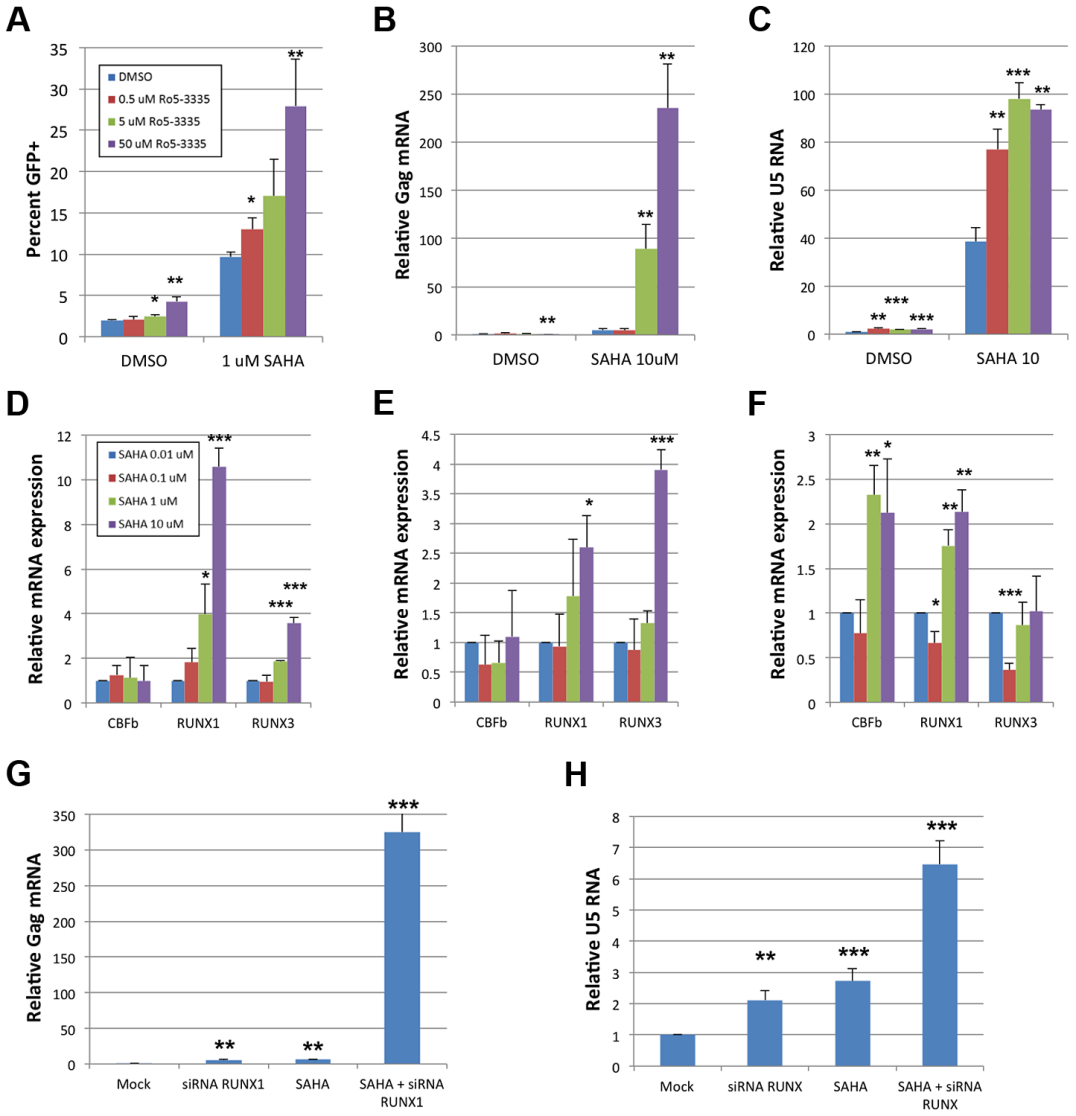
Ro5-3335 RUNX inhibitor synergizes with SAHA HDAC inhibitor. **A**) JLat cells were cultured in the presence of increasing concentrations of Ro5-3335 in the presence or absence of 1 μM SAHA. Seventy-two hours after treatment the percentage of GFP positive cells was determined by flow cytometry. **B**) ACH2 cells were cultured with Ro5-3335 and 10 μM SAHA as in panel A. Forty-eight hours post treatment RNA was extracted from the cells and used to determine the relative increase in HIV-1 Gag mRNA as normalized to GAPDH. **C**) TZMbl cells were cultured with Ro5-3335 and 10 μM SAHA as above. Forty-eight hours post treatment RNA was extracted from the cells and used to determine the relative increase in HIV-1 U5 mRNA as normalized to GAPDH. **D**) Jlat, **E**) ACH2 and **F**) TZMbl were cultured in the presence of the indicated concentrations of SAHA. Forty-eight hours post treatment RNA was extracted from the cells and used to determine the relative changes in RUNX1, CBF-β or RUNX3. **G**) ACH2 or **H**) TZMbl cells were transfected with 50 pMol siRNA against RUNX1 and/or treated with 10 μM SAHA at twenty-four hours post transfection. Forty-eight hours post transfection RNA was extracted and expression measured as above. * p≤0.05, ** p≤0.01 and *** p≤0.001

The benzodiazepine compound Ro5-3335 was recently identified as an inhibitor of RUNX1:CBF-β interaction and their functions in transcriptional regulation and hematopoiesis [24]. Suppression of RUNX1 or CBF-β by siRNA was capable of reactivating latent cells, suggesting that a pharmacologic inhibitor of RUNX1/CBF-β function may have a similar effect (Supplementary Figure S2). To test this hypothesis the Jlat model of HIV-1 latency [36] was used wherein viral reactivation can be followed by GFP expression. Jlat cells were treated with DMSO, 0.5, 5 or 50 μM Ro5-3335 for 72 hours and re-activation was assayed by flow cytometry (Fig 6A). Treatment with 50 uM Ro5-3335 induced a 2.2-fold increase in the number of GFP positive cells (1.9 to 4.2%). This two-fold activation is similar in magnitude to the effect seen with siRNA (Supplementary Figure S2). We next tested if the effect of Ro5-3335 could be increased by treatment with a second drug. For this purpose, we chose the HDAC inhibitor suberoylanilide hydroxamic acid (SAHA), also known as Vorinostat, which has previously been used to reactivate latently infected T-cells in HIV-1 patients. Treatment of Jlat cells with 1 μM SAHA increased the number of GFP positive cells by 5.1-fold (1.9 to 9.6%) – similar to the induction of RT activity in ACH2 (Supplementary Figure S5). Treatment with increasing concentrations of Ro5-3335 increased the percentage of GFP positive cells in a multiplicative fashion. Treatment with 50 uM Ro5-3335 was capable of activating another 2.9-fold above SAHA alone, for a total activation of 28%. The combination of Ro5-3335 and SAHA yielded similar results in ACH2 (Fig 6B) and TZMbl (Fig 6C) cell lines. Of potential concern to the development of any therapeutic is the issue of toxicity. To address this we treated JLat, ACH2, TZMbl and J-LTR-G (a Jurkat cell line that carries an integrated LTR-GFP reporter) with SAHA and Ro5-3335 (Supplementary Figure S6). Ro5-3335 alone had minimal effects on cell viability. Treatment with SAHA induced significant cell death in all four cell types. Treatment of JLat, ACH2, TZMbl and J-LTR-G with SAHA and increasing concentrations of Ro5-3335 induced further cell death beyond SAHA alone (Fig S5, compare final four data sets for panels A–D). Interestingly, JLat and ACH2 had the greatest cell death (15% and 12% live when treated with SAHA and 50 uM Ro5-3335) as compared to TZMbl and J-LTR-G (50% and 44%). JLat and ACH2 also experienced greater cell death with SAHA treatment alone (55% and 51% live vs 81% and 63% live). ACH2 and JLat both carry complete copies of the virus, whereas TZMbl and J-LTR-G are reporter only cell lines. It is tempting to posit that the increased cell death seen in ACH2 and JLat is due to reactivation of the virus. The multiplicative effect on HIV reactivation seen with Ro5-3335 and SAHA in the three cell lines tested is indicative of possible synergy.

True synergy implies that two compounds are working on the same pathway or mechanism. There are two likely points of interaction that would cause synergy between Ro5-3335 and SAHA. RUNX proteins are capable of recruiting HDACs [19] and this may be happening at the HIV-1 promoter. Alternatively, the inhibition of HDACs by SAHA will induce broad changes in gene expression and this may include changes in RUNX1 and CBF-β expression. To test the later point, we treated cells with SAHA and measured the expression of CBF-β, RUNX1 and RUNX3 by quantitative RT-PCR (Fig 6D–F). Treatment of Jlat with SAHA triggered increased expression of RUNX1 and RUNX3 (Panel D) and a similar pattern was observed for ACH2 (Panel E). Treatment of TZMbl with SAHA also induced RUNX1 expression and induced CBF-β as well (Panel F).

To further verify that the observed synergy of SAHA and Ro5-3335 is due to the involvement of RUNX1 we asked if siRNA knockdown of *RUNX1* would synergize with SAHA in the same way as the drug. ACH2 (Fig 6G) and TZMbl (Fig 6H) cells were again transfected with 50 pMol of siRNA against *RUNX1* and then treated with either DMSO or 10 μM SAHA at 24 hours post transfection. Twenty-four hours post drug-treatment RNA was extracted from the cells and activation was measured by quantitative RT-PCR against Gag (ACH2) or U5 (TZMbl). Treatment of ACH2 with siRNA alone induced a 4.8-fold increase in expression. siRNA treatment reduced expression of RUNX1 and CBF-β by 52% and 63% respectively. Treatment with SAHA induced a 6.2-fold increase. Treatment with both siRNA against RUNX1 and SAHA led to a greater than 300 fold activation. In TZMbl cells, a similar, but smaller effect was seen. Treatment with siRNA or SAHA alone induced a 2.1 and 2.7-fold increase respectively. Treatment of TZMbl with both siRNA against RUNX1 and SAHA yielded a 6.5-fold increase in transcription. These results are consistent with that seen with Ro5-3335 and SAHA and strongly suggest that RUNX1/CBF-β inhibition may improve the ability of SAHA to activate HIV-1.

**Figure 7:**
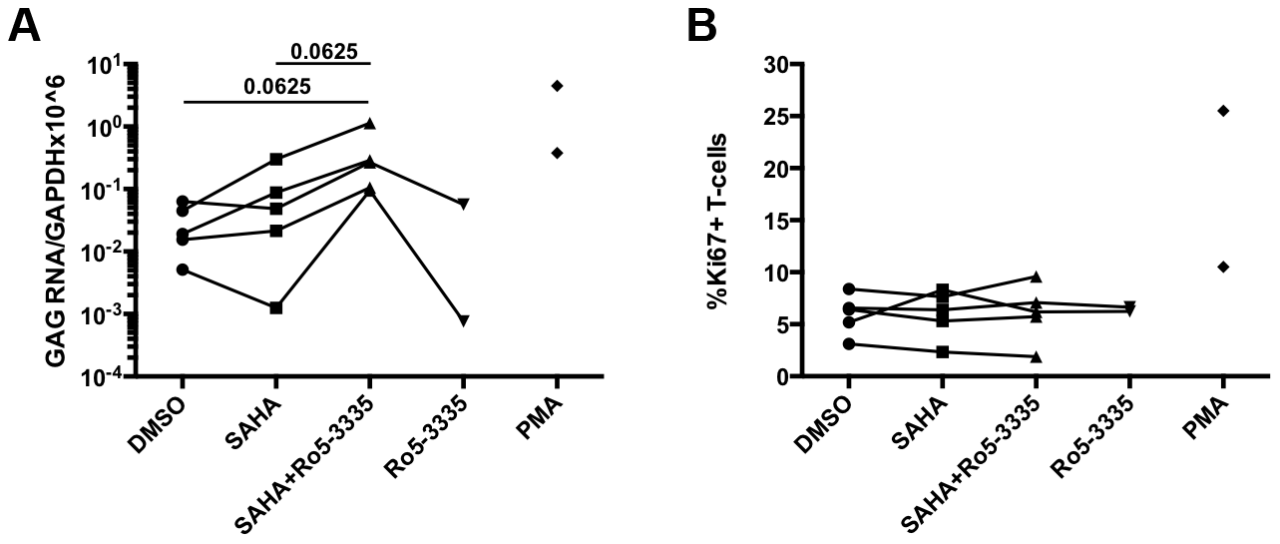
Ro5-3335 improves re-activation of HIV-1 in patient samples. PBMCs from HIV-1 patients suppressed on therapy were treated with DMSO, 250 nM SAHA, 5 uM Ro5-3335 or both drugs in combination. PMA treatment was used as a positive control. **A**) Twenty-four hours after treatment RNA was isolated from cells and used to detect Gag mRNA by RT-qPCR. **B**) Flow cytometry was used to determine the percentage of activated T-cells by staining for intracellular Ki67.

The elucidation of RUNX1 involvement in HIV-1 latency described above is heavily dependent upon cell line models of latency. Although these minimalistic systems are useful in determining the possible mechanism of action of a given protein or pathway, they do not represent an ideal model system for evaluating potential therapeutic intervention. The gold standard for evaluating latency is the ability to reactivate latent virus from the PBMCs of HIV-1 patients on suppressive therapy. In order to evaluate the effect of Ro5-3335 in primary cells we obtained PBMCs from five HIV-1 patients on suppressive therapy who had undetectable viral loads for at least 6 months. PBMCs were then treated with SAHA and Ro5-3335 to induce reactivation. In brief, 10×10^6^ PBMCs were divided as needed for multiple conditions and placed in 10 ml RPMI +10%FBS. Cells from one patient were treated with 250 nM SAHA, 250 nM SAHA plus 5 uM Ro5-3335 or DMSO control. A SAHA dose of 250 nM was chosen as it has been shown to be equivalent to the concentration of available drug in the sera of patients [37]. Cells from two patients were treated with the above combinations plus 5 uM Ro5-3335 alone. Finally, cells from the final two patients were incubated with the three conditions plus 1 uM phorbol myristate acetate (PMA). Twenty-four hours after treatment the cells were collected, a small portion (∼300 k cells) was used for flow cytometry to detect activation of T-cells by Ki67 and cell death by vital stain. RNA was extracted from the remaining cells and used for RT-qPCR to detect HIV Gag mRNA (Fig 7A). The background level of Gag mRNA varied from 0.005 to 0.0633 copies/10^6^ GAPDH. In three of the five patient samples treatment with SAHA induced a noticeable increase in Gag mRNA ranging from 1.4 to 6.7-fold. In all five patient samples treatment with SAHA and Ro5-3335 increased the levels of Gag mRNA beyond the levels seen in SAHA treatment (from 3.2 to 75-fold). A Wilcoxon matched pairs test showed a p-value that was approaching significance (p = 0.0625). Control cultures treated with 5 uM Ro5-3335 alone showed no increase in activation as compared to control. Flow cytometric analysis of the cells revealed no increase in T-cell activation (Fig 7B) or cell death (Supplementary Figure S5E).

## Discussion

In this study, we report a role for the RUNX family of transcription factors in repressing HIV-1 transcription driven by the viral LTR. The RUNX1 protein is involved in fate determination of T-cells and control of CD4 expression [12], [14], [38], [39] making its potential involvement in HIV replication of physiological interest. We have identified interaction of RUNX1 and the co-factor CBF-β with the viral LTR through a potential binding site (Fig 3) and that alteration of RUNX1 and CBF-β expression alters viral replication (Fig 1).

Work in in the field of HIV Vif biology has identified a role for CBF-β in Vif mediated degradation of APOBEC3G [21], [22]. We have uncovered new mechanisms for the involvement of CBF-β in HIV infection in the context of Vif expression, demonstrating that another functional consequence of Vif:CBF-β interaction is rescue of the virus from transcriptional repression by RUNX1 (Fig 4). A Vif mutant lacking the region necessary for CBF-β binding [20], [22] is incapable of counteracting RUNX1 repression of transcription, thus codifying the role of Vif in protecting the viral promoter.

Perhaps most interestingly we show that repression of RUNX1 activity by a pharmacologic inhibitor (Ro5-3335) is capable of synergizing with the HDAC inhibitor SAHA (Fig 6 and 7). True synergy implies that two compounds are working on the same pathway or mechanism. There are two likely points of interaction that would cause synergy between Ro5-3335 and SAHA. RUNX proteins are capable of recruiting HDACs [19] and this may be happening at the HIV-1 promoter. Alternatively, the inhibition of HDACs by SAHA will induce broad changes in gene expression and this may include changes in RUNX1 and CBF-β expression. Blocking RUNX1 function during SAHA treatment (via either siRNA or drug) significantly increases the activity of SAHA on the LTR. SAHA has been the recent drug of choice employed in studies attempting to reactivate latently infected memory T-cells – a necessary step in clearing the latent reservoir [37], [40]–[43]. Although the drug potently activates cells in culture and has a measurable effect on viral transcription in the T-cells of patients, it has not been successful in reducing the percentage of infected memory cells [44]. Combination therapy in which SAHA is delivered alongside a RUNX inhibitor may provide greater activation of virus, which in turn could lead to greater cell death or greater response by anti-HIV CD8+ T-cells.

It is of note that Ro5-3335 and a related drug have crossed the HIV literature before. A 1991 screen by Hsu *et al.* identified Ro5-3335 as a potential inhibitor of HIV-1 transcription [45]. However, the exact mechanism of this inhibition is unknown and several follow up studies paint a very complex picture. Another analysis of potential Tat inhibitors determined that Ro5-3335 did not inhibit Tat-TAR interaction [46]. We found recently that Tat binds RUNX1 with high affinity and inhibits Tat-mediated transcription together with CBF-β [24]. Combined with data presented here, our findings suggest that RUNX1 potentially influences Tat transactivation, and is the true target of Ro5-3335.

An analysis of Ro5-3335 and a related drug (Ro 24-7429) determined that they had no suppressive effect in chronically infected cells [47]. A later analysis by Cupelli and Hsu concluded that the drug might act at the level of initiation [48]. A clinical trial using Ro 24-7429 failed to reduce viral load and the presence of infectious virus in patient plasma, or to increase CD4 T-cell count [49]. Indeed, in our hands the activation of HIV-1 by Ro5-3335 happens in a timeframe beyond what was examined in the initial studies. This may explain why no inhibition was seen in chronically infected cells and suggests that the drug acts in a positive fashion on a fully integrated and chromatinized promoter.

What remains unanswered is how RUNX proteins might shape the clinical outcomes during HIV infection. Analysis of CD4+ T-cell populations (Supplementary Figure S4) shows that upon activation of naïve CD4+ T-cells (a population relatively refractory to infection) the expression levels of RUNX1, RUNX3 and CBF-β drop (correlating with greater susceptibility to infection). These findings are similar to what has been demonstrated in mice [13], [50], [51], although the detection of RUNX3 in CD4+ T-cells suggests a possible difference between humans and rodents. Interestingly, the expression of these proteins in naïve cells is fairly uniform, but expression in the memory pool (the primary target cells during infection) is much more variable. Although microarray databases show general expression of RUNX1 and CBF-β across tissues [52], no definitive studies have yet been performed in human cells which examine expression in specific T-cell subsets. Data presented in figure 5 shows a significant negative correlation between RUNX1 expression and viral load, as well as a significant positive correlation between RUNX1 expression and CD4+ T-cell counts in the absence of therapy. However, further research is needed to identify the causal relationship that drives this correlation.

## Materials and Methods

### Ethics statement

The human sample collection protocol was approved by the NIH Clinical Center Institutional Review Board as part of a separate ongoing study. Written informed consent was obtained in all cases and all applicable protections of patient rights and privacy applied. For this study specific samples were requested from the sample bank based on given criteria.

### Cell culture and transfections

Adherent cell lines (293T and TZMbl) were maintained in DMEM supplemented with 10% fetal bovine serum, L-glutamine and penicillin/streptomycin. 293T and TZMbl cells were transfected using Lipofectamine LTX (Invitrogen) according to the manufacturer's instructions.

Suspension cell lines (Jurkat, Jlat, J-LTR-G and ACH2) were maintained in RPMI supplemented with 10% fetal bovine serum, L-glutamine and penicillin/streptomycin. Jurkat and ACH2 cell lines were transfected using the Amaxa Nucleofector (Lonza) with reagent kit V and programs X-005 or T-014 respectively.

### Chromatin immunoprecipitation

Cells for chromatin immunoprecipitation (ChIP) were cross-linked using 1% formaldehyde for 10 minutes at 37°C. Following crosslinking, cells were lysed in 1% SDS, 10 mM EDTA and 50 mM Tris-HCl pH 8.1 and sheared by sonication to less than 1000 bp. Lysate was clarified by centrifugation and diluted 1∶10 with 0.01% SDS, 1.1% Triton X-100, 1.2 mM EDTA, 16.7 mM Tris-HCl and 167 mM NaCl for antibody incubation. Following overnight incubation with antibody, complexes were precipitated using protein A/G agarose beads and washed with low salt (0.1% SDS, 1% Triton X-100, 2 mM EDTA, 20 mM Tris-HCl and 150 mM NaCl), high salt (0.1% SDS, 1% Triton X-100, 2 mM EDTA, 20 mM Tris-HCl and 500 mM NaCl) and lithium chloride (0.25 M LiCl, 1% NP40, 1% deoxycholate, 1 mM EDTA and 10 mM Tris-Hcl) buffers. Complexes were eluted in 1% SDS, 0.1 M NaHCO3 and reverse crosslinked by overnight incubation at 65°C. Proteinase K was used to digest remaining protein before DNA was cleaned using phenol:chloroform and precipitated prior to qPCR with primers for the LTR (174-464) or Gag (bp 1852-2153). Data is represented as signal for each primer pair relative to total input DNA (before IP).

Although positive signal in this LTR primer set might suggest that the relevant RUNX binding site is located between base pairs 174-464, the chromatin used in this analysis was sheared to less than 1000 bases meaning that the binding could be anywhere within the LTR.

### Site-directed mutagenesis

RUNX binding site mutants were created in pLTR-Luc using QuikChange XL site directed mutagenesis kit (Stratagene) and primer pairs RUNXMut2, RUNXMut5, RUNXMut6 and RUNXMut7 (see below). Mutants were verified by sequencing.

### Confocal microscopy

HeLa CBF-β knockdown (KD) cells (5×10^6^) were transfected with the Vif expression vector pNL-A1 (2.5 μg), the CBF-β vector pCBF-β (1 μg) or were cotransfected with both vectors (2.5∶1 plasmid ratio). Total amounts of transfected DNA were adjusted to 5 μg using empty vector DNA as appropriate. Three hours after transfection, cells were trypsinized and seeded onto cover slips. Cells were fixed 24 hr later in methanol (10 min, −20°C) and then stained with rabbit antibodies to Vif (Vif93; 1∶100) or CBF-β (Thermo Fisher; 1∶100). Nuclear membranes were stained with a mouse monoclonal antibody to lamin B (RDI; 1∶100). Bound antibodies were visualized by Texas-Red or Cy2-conjugated secondary antibodies (Jackson Labs; 1∶100). Images were collected on a Zeiss LSM410 confocal microscope using a Plan-Apochromat 63x/1.4 oil immersion objective (Zeiss).

### Flow cytometry and primary cell analysis

To analyze the expression levels of RUNX1 and CBF-β in primary human T-cells, I sorted resting and activated naïve and memory CD4+ and CD8+ T-cells from PBMCs. In brief, PBMC were cultured overnight in the presence of absence of SEB to broadly activate T-cells. Flow cytometry was then used to sort CD3+ T-cells into different populations. Resting cells in the unstimulated population were identified as CD69- and activated cells from the SEB treatment as CD69+. Cells were then further classed as naïve (CD27+, CD45RO−) or memory (CD45RO+, CD27−). Finally, T-cell populations were sorted by the presence of CD4 or CD8 T-cell co-receptor. For the reactivation work, cells were analyzed for cell death using Live/Dead fixable Aqua (Life Technologies) and stained for CD3 and Ki67. Populations of T-cells were used to prepare RNA using Trizol reagent and RNA was submitted for RT-qPCR for RUNX1.

### Statistical analysis

Data represented in graphical form is always the average of at least three replicates with standard deviation. Statistical significance was determined using an unpaired Student's t-test (cell culture) Mann-Whitney (Primary cells) or a Spearman's exact test with a cutoff of p<0.05.

**Primers.** HIV U5 F CTGCATGGGATGGAGGA

HIV U5 R GTTAGCCAGAGAGCTCCCAG

HIV Gag2 F GGTGCGAGAGCGTCAGTATTAAG

HIV Gag2 R AGCTCCCTGCTTGCCCATA

RUNXMut2 F atccttgatctgtggatctcacacacacaaggctacttcc

RUNXMut2 R ggaagtagccttgtgtgtgtgagatccacagatcaaggat

RUNXMut5 F CCAGGGAGGTGTGTGCTGGGCGGGACTG

RUNXMut5 R CAGTCCCGCCCAGCACACACCTCCCTGG

RUNXMut6 F CTGTACTGGGTCTCTCTGTGTAGACCAGATCTGAGCCT

RUNXMut6 R AGGCTCAGATCTGGTCTACACAGAGAGACCCAGTACAG

RUNXMut7 F GGGTCTCTCTGGTTAGCACAGATCTGAGCCTGGG

RUNXMut7 R CCCAGGCTCAGATCTGTGCTAACCAGAGAGACCC

GAPDH F GCTCACTGGCATGGCCTTCCGTGT

GAPDH R TGGAGGAGTGGGTGTCGCTGTTGA

RUNX1 F GATGGCACTCTGGTCACTGTGA

RUNX1 R CTTCATGGCTGCGGTAGCAT

RUNX3 F TTCCTAACTGTTGGCTTTCC

RUNX3 R TAGGTGCTTTCCTGGGTTTA

CBF-β F ACAGCGACAAACACCTAGCC

CBF-β R CAGCCCATACCATCCAGTCT

RUNX1 Proximal F TGCATGATAAAAGTGGCCTTGT

RUNX1 Proximal R CGAAGAGTAAAACGATCAGCAAAC

RUNX1 Distal F TGGTTTTCGCTCCGAAGGT

RUNX1 Distal R CATGAAGCACTGTGGGTACGA

## Supporting Information

Figure S1 **Over-expression of RUNX1 and CBF-β in Jurkat T-cells.** Jurkat T-cells were transfected with 1 ug of pMAXGFP or 0.04, 0.2 or 1 ug of pRUNX1 or pCBF-β. Forty-eight hours after transfection the cells were collected, lysed and used for Western blotting to detect RUNX1, CBF-β or β-actin.
(TIFF)

Figure S2 **Knockdown of RUNX1 or CBF-β re-activates virus from latency.** ACH2 latently infected T-cell line was transfected with 2, 10 or 50 pMol siRNA against RUNX 1 or CBF-β. **A)** Supernatant was harvested from ACH2 cell cultures 48 hours post transfection and used to determine viral production by RT assay. RT-qPCR was performed on RNA extracted from the cells at 48 hours to determine the expression levels of **B)** RUNX1 or **C)** CBF-β. * p≤0.05, ** p≤0.01 and *** p≤0.001.
(TIFF)

Figure S3 **RUNX1 and CBF-β effect on control promoters.** Control transfections were performed using the **A)** MMLV BXH2 promoter (positive control) and **B)** EF1 and **C)** CMV promoters. One microgram of reporter construct was transfected into 293T cells alongside 1 ug of RUNX1 or CBF-β expression vectors or a combination of the two. Mass of DNA was held constant using an empty vector. 48 hours post-transfection the cells were harvested and used to determine the expression of luciferase or GFP as appropriate.
(TIFF)

Figure S4 **Expression of RUNX proteins and CBF-β in primary T-cell subsets.** PBMCs from healthy donors were cultured overnight in the presence or absence of SEB. Flow cytometry was then used to sort CD3+ T-cells into different populations. Resting cells in the unstimulated culture were identified as CD69- and activated cells from the SEB treatment as CD69+. Cells were then further classed as naïve (CD27+ CD45RO-) or memory (CD45RO+ CD27-). Finally, T-cell populations were sorted by the presence of CD4 or CD8 T-cell co-receptor. RNA was prepared using Trizol reagent and submitted for RT-qPCR for RUNX1, CBF-β and RUNX3. **A)** Expression of RUNX1, CBF-β and RUNX3 in resting naïve and memory CD4+ T-cells from four healthy donors. The variance (s2) was determined for each data set. **B)** Effect of activation on expression levels in naive and **C)** memory CD4+ T-cells. ** p≤0.01 and *** p≤0.001**.**
(TIFF)

Figure S5 **Reactivation with SAHA, ValproA or Ro5-3335.** ACH2 cells were treated with increasing concentrations of PMA (0, 1, 10 uM), SAHA (0, 1, 10 uM), ValproA (0, 1, 10 mM) and Ro5-3335 (0, 5 and 50 uM). Forty-eight hours post transfection viral production was determined by RT assay on the cell culture supernatant.
(TIFF)

Figure S6 **Toxicity of SAHA and Ro5-3335.** **A)** JLat, **B)** ACH2, **C)** TZMbl or **D)** J-LTR-G were cultured with increasing concentrations of Ro5-3335 and SAHA as indicated. Forty-eight hours post treatment cells were stained with Trypan Blue and cell the percentage of live cells was determined by light microscopy. **E)** PBMC from patients suppressed on therapy were treated with 250 nM SAHA, 5 uM Ro5-3335 or a combination of the two. Twenty-four hours after treatment the cells were collected and percentage of live cells was determined by flow cytometry using a vital stain.
(TIFF)
