# Supplementary figures and images for: Activation of HIV-1 from Latent Infection via Synergy of RUNX1 Inhibitor Ro5-3335 and SAHA
Source: PLoS Pathog. 2014 Mar 20;10(3):e1003997. doi: 10.1371/journal.ppat.1003997 (PMC3961356; doi:10.1371/journal.ppat.1003997)

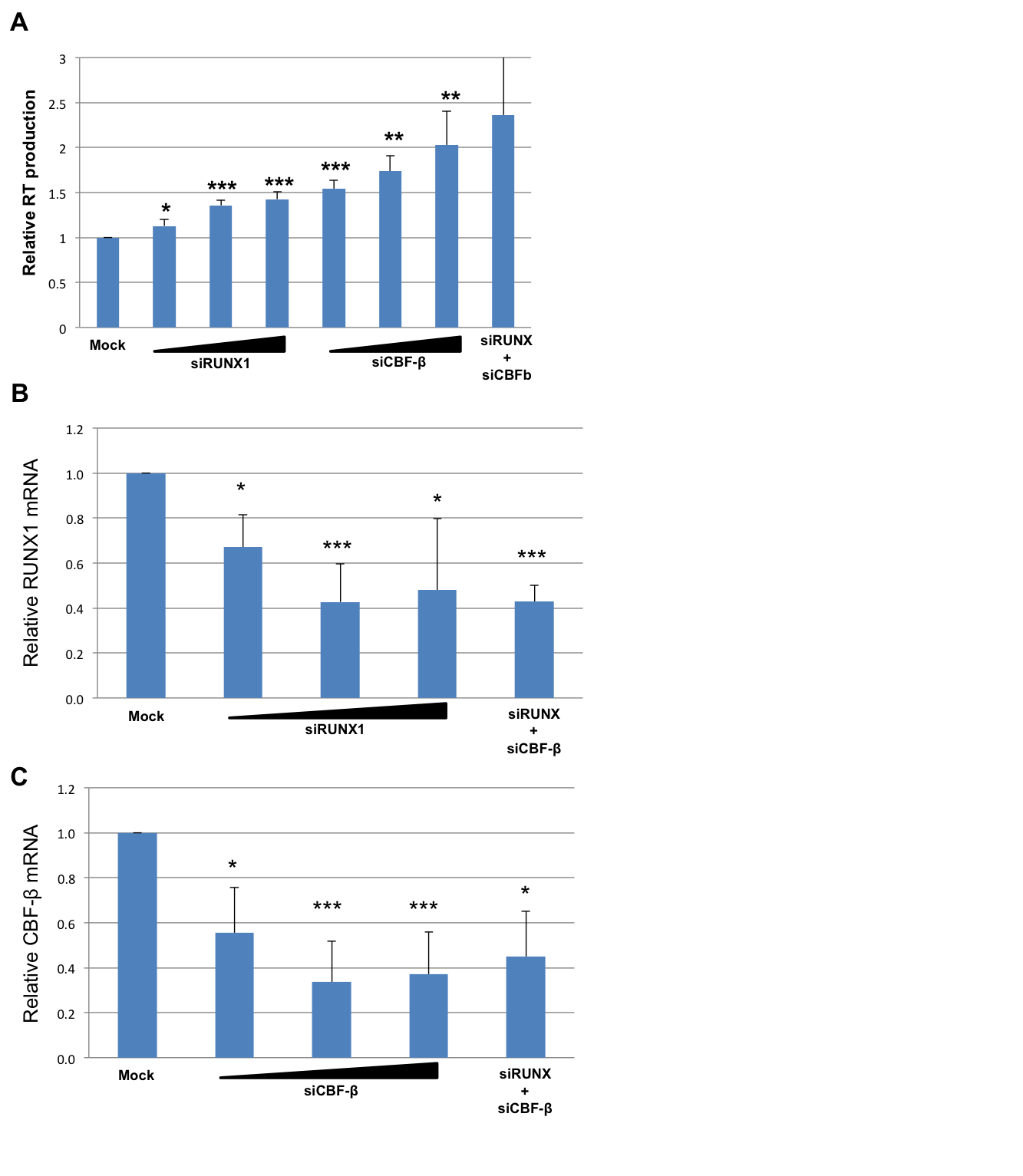

Supplement: Figure S2 — Knockdown of RUNX1 or CBF-β re-activates virus from latency. ACH2 latently infected T-cell line was transfected with 2, 10 or 50 pMol siRNA against RUNX 1 or CBF-β. A) Supernatant was harvested from ACH2 cell cultures 48 hours post transfection and used to determine viral production by RT assay. RT-qPCR was performed on RNA extracted from the cells at 48 hours to determine the expression levels of B) RUNX1 or C) CBF-β. * p≤0.05, ** p≤0.01 and *** p≤0.001. (TIFF) [file ppat.1003997.s002.tiff]

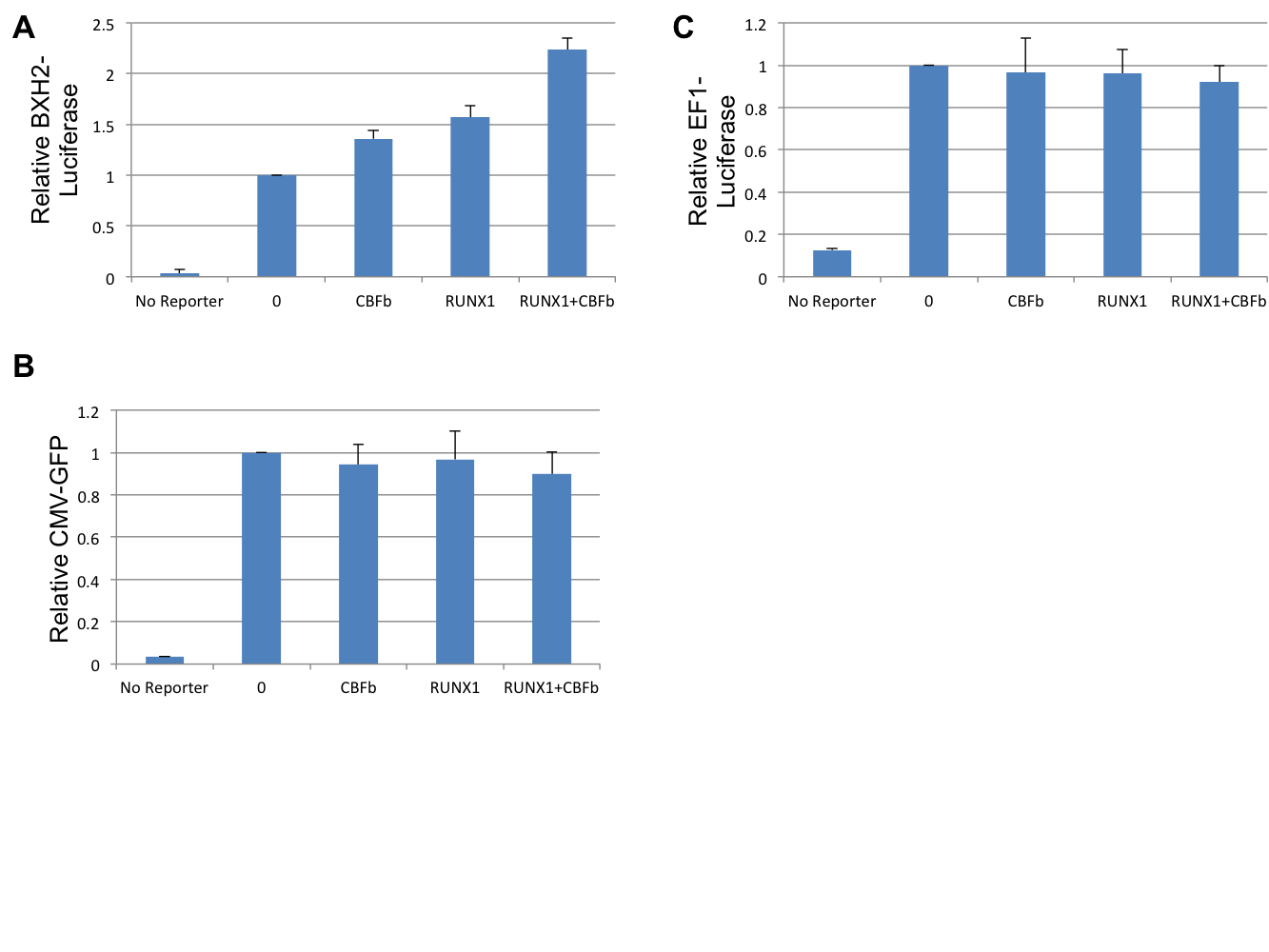

Supplement: Figure S3 — RUNX1 and CBF-β effect on control promoters. Control transfections were performed using the A) MMLV BXH2 promoter (positive control) and B) EF1 and C) CMV promoters. One microgram of reporter construct was transfected into 293T cells alongside 1 ug of RUNX1 or CBF-β expression vectors or a combination of the two. Mass of DNA was held constant using an empty vector. 48 hours post-transfection the cells were harvested and used to determine the expression of luciferase or GFP as appropriate. (TIFF) [file ppat.1003997.s003.tiff]

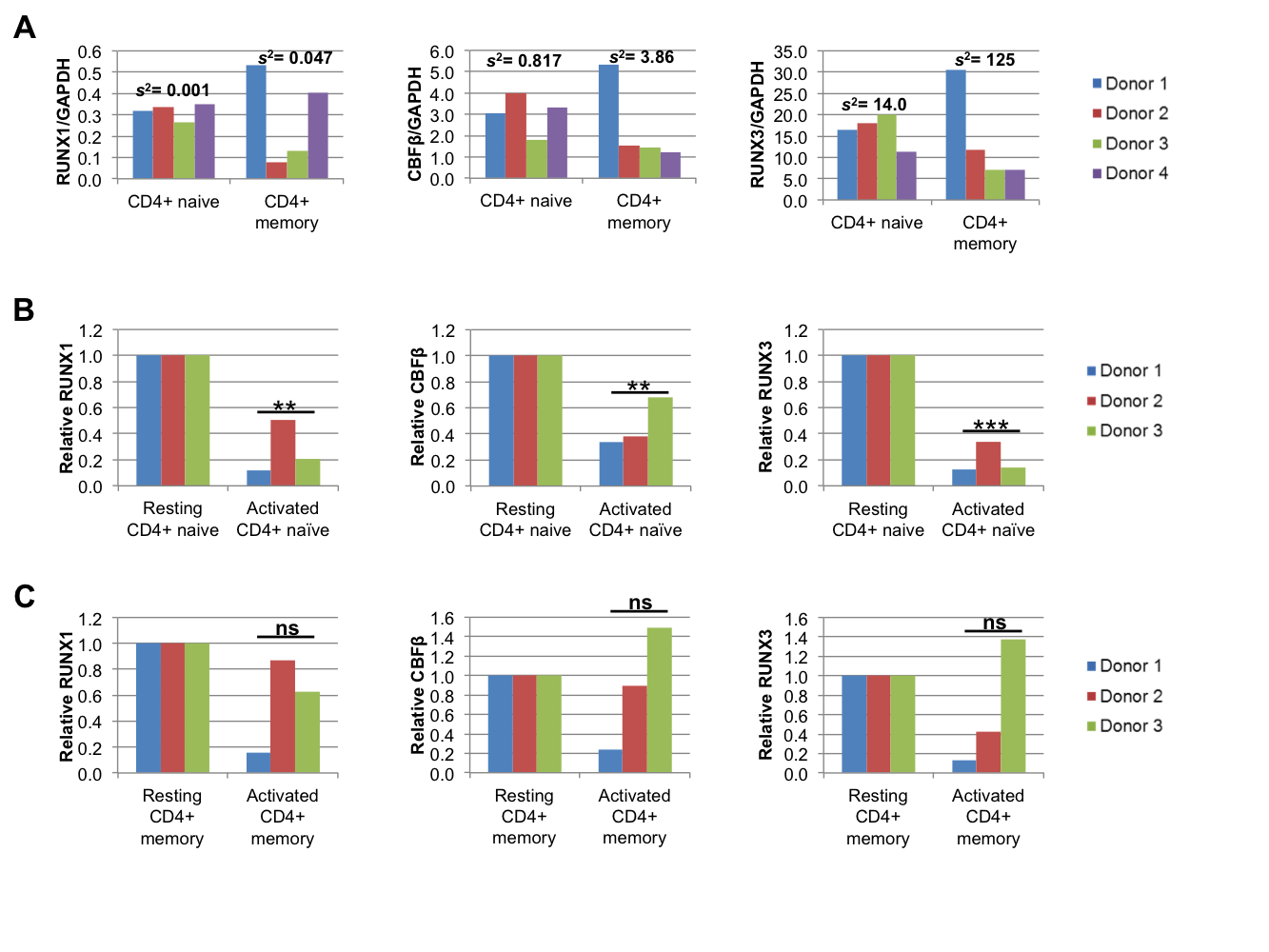

Supplement: Figure S4 — Expression of RUNX proteins and CBF-β in primary T-cell subsets. PBMCs from healthy donors were cultured overnight in the presence or absence of SEB. Flow cytometry was then used to sort CD3+ T-cells into different populations. Resting cells in the unstimulated culture were identified as CD69- and activated cells from the SEB treatment as CD69+. Cells were then further classed as naïve (CD27+ CD45RO-) or memory (CD45RO+ CD27-). Finally, T-cell populations were sorted by the presence of CD4 or CD8 T-cell co-receptor. RNA was prepared using Trizol reagent and submitted for RT-qPCR for RUNX1, CBF-β and RUNX3. A) Expression of RUNX1, CBF-β and RUNX3 in resting naïve and memory CD4+ T-cells from four healthy donors. The variance (s2) was determined for each data set. B) Effect of activation on expression levels in naive and C) memory CD4+ T-cells. ** p≤0.01 and *** p≤0.001. (TIFF) [file ppat.1003997.s004.tiff]

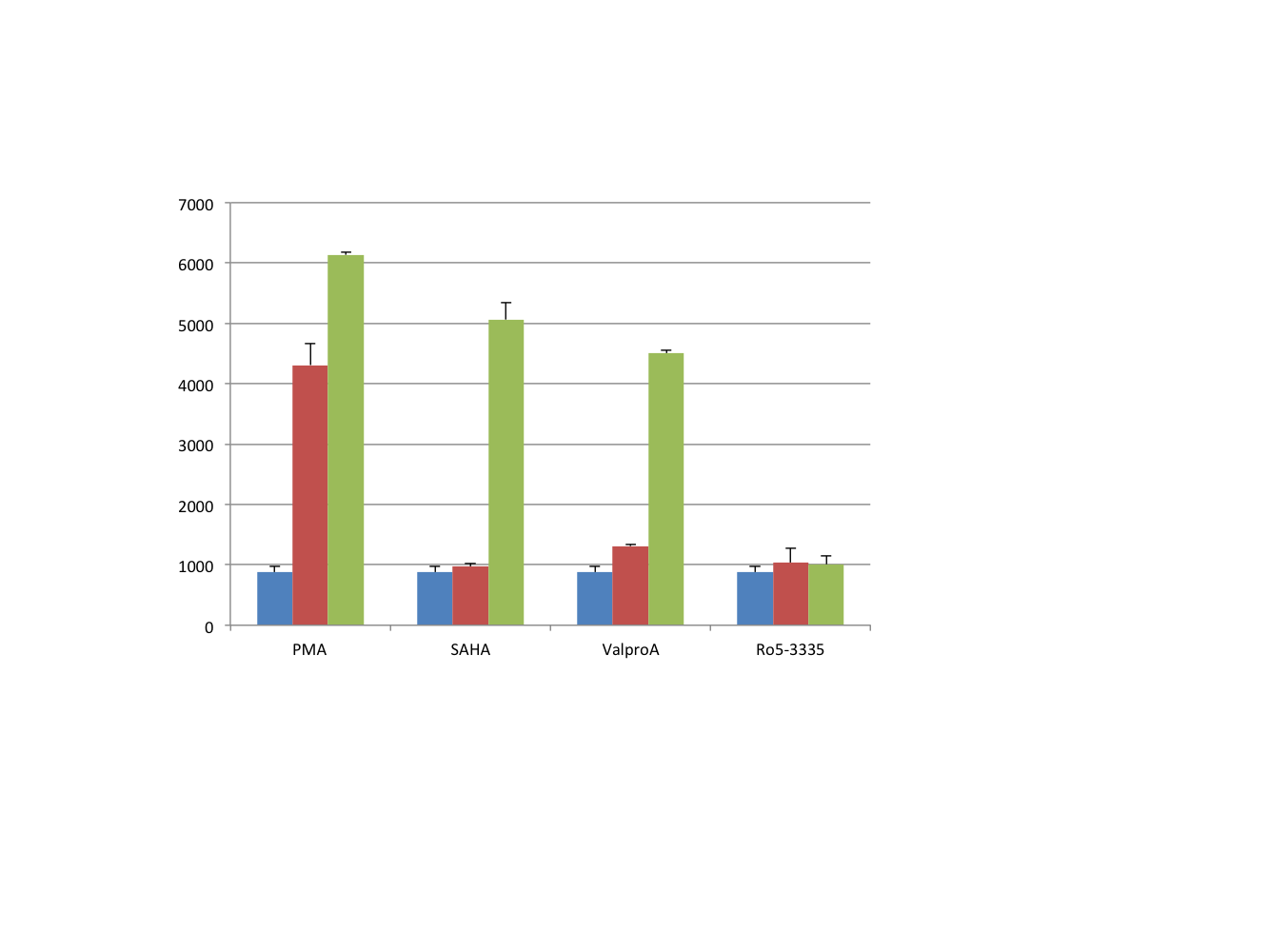

Supplement: Figure S5 — Reactivation with SAHA, ValproA or Ro5-3335. ACH2 cells were treated with increasing concentrations of PMA (0, 1, 10 uM), SAHA (0, 1, 10 uM), ValproA (0, 1, 10 mM) and Ro5-3335 (0, 5 and 50 uM). Forty-eight hours post transfection viral production was determined by RT assay on the cell culture supernatant. (TIFF) [file ppat.1003997.s005.tiff]

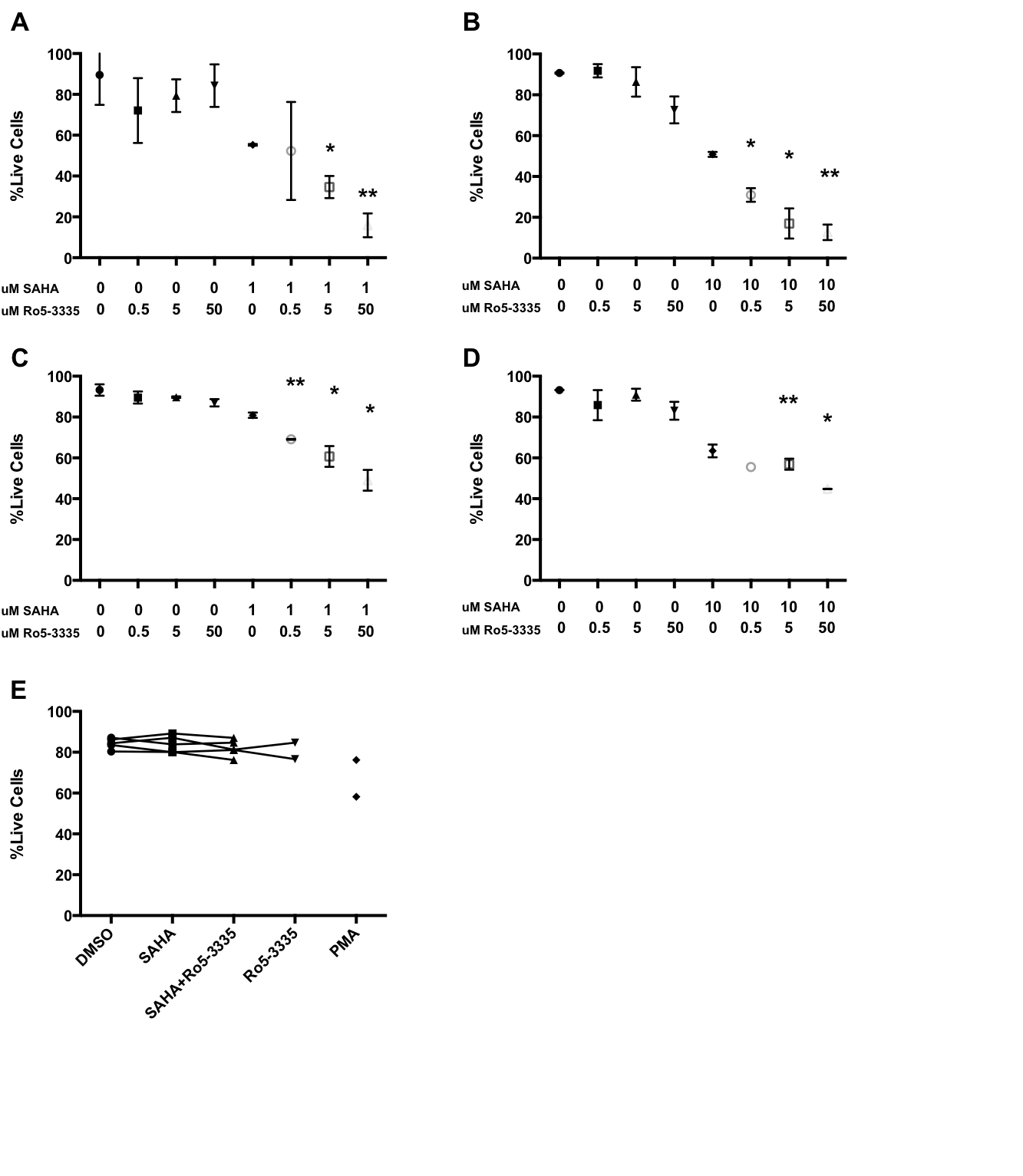

Supplement: Figure S6 — Toxicity of SAHA and Ro5-3335. A) JLat, B) ACH2, C) TZMbl or D) J-LTR-G were cultured with increasing concentrations of Ro5-3335 and SAHA as indicated. Forty-eight hours post treatment cells were stained with Trypan Blue and cell the percentage of live cells was determined by light microscopy. E) PBMC from patients suppressed on therapy were treated with 250 nM SAHA, 5 uM Ro5-3335 or a combination of the two. Twenty-four hours after treatment the cells were collected and percentage of live cells was determined by flow cytometry using a vital stain. (TIFF) [file ppat.1003997.s006.tiff]
